# Hypoacetylation, hypomethylation, and dephosphorylation of H2B histones and excessive histone deacetylase activity in DU-145 prostate cancer cells

**DOI:** 10.1186/s13045-016-0233-x

**Published:** 2016-01-12

**Authors:** Shundong Cang, Xiaobin Xu, Yuehua Ma, Delong Liu, J. W. Chiao

**Affiliations:** Department of Oncology, The People’s Hospital of Henan Province, Zhengzhou, Henan 450052 China; Department of Medicine, New York Medical College, Valhalla, NY 10595 USA; Center for Biomedical Mass Spectrometry, Boston University School of Medicine, Boston, MA 02118 USA; Present address: Regeneron Pharmaceuticals, Inc., Tarrytown, NY 10591 USA

**Keywords:** Epigenomes, Histone H2B modifications, Mass spectrometry, Acetylation, Deacetylases, Hypoacetylation, Prostate cancer

## Abstract

**Background:**

Hypoacetylation on histone H3 of human prostate cancer cells has been described. Little is known about the modifications of other histones from prostate cancer cells.

**Methods:**

Histones were isolated from the prostate cancer cell line DU-145 and the non-malignant prostatic cell line RC170N/h. Post-translational modifications of histone H2B were determined by liquid chromatography-mass spectrometry (LC-MS)/MS.

**Results:**

The histone H2B of the prostate cancer cell line DU-145 was found to have hypoacetylation, hypomethylation, and dephosphorylation as compared to the non-malignant prostatic cell line RC170N/h. H2B regained acetylation on multiple lysine residues, phosphorylation on Thr19, and methylation on Lys23 and Lys43 in the DU-145 cells after sodium butyrate treatment.

**Conclusions:**

The histone H2B of DU-145 prostate cancer cells are hypoacetylated, hypomethylated, and dephosphorylated. Histone deacetylase inhibitor reversed this phenotype. Epigenetic agent may therefore be useful for prostate cancer therapy and worth further investigation.

**Electronic supplementary material:**

The online version of this article (doi:10.1186/s13045-016-0233-x) contains supplementary material, which is available to authorized users.

## To the editor

**Fig. 1:**
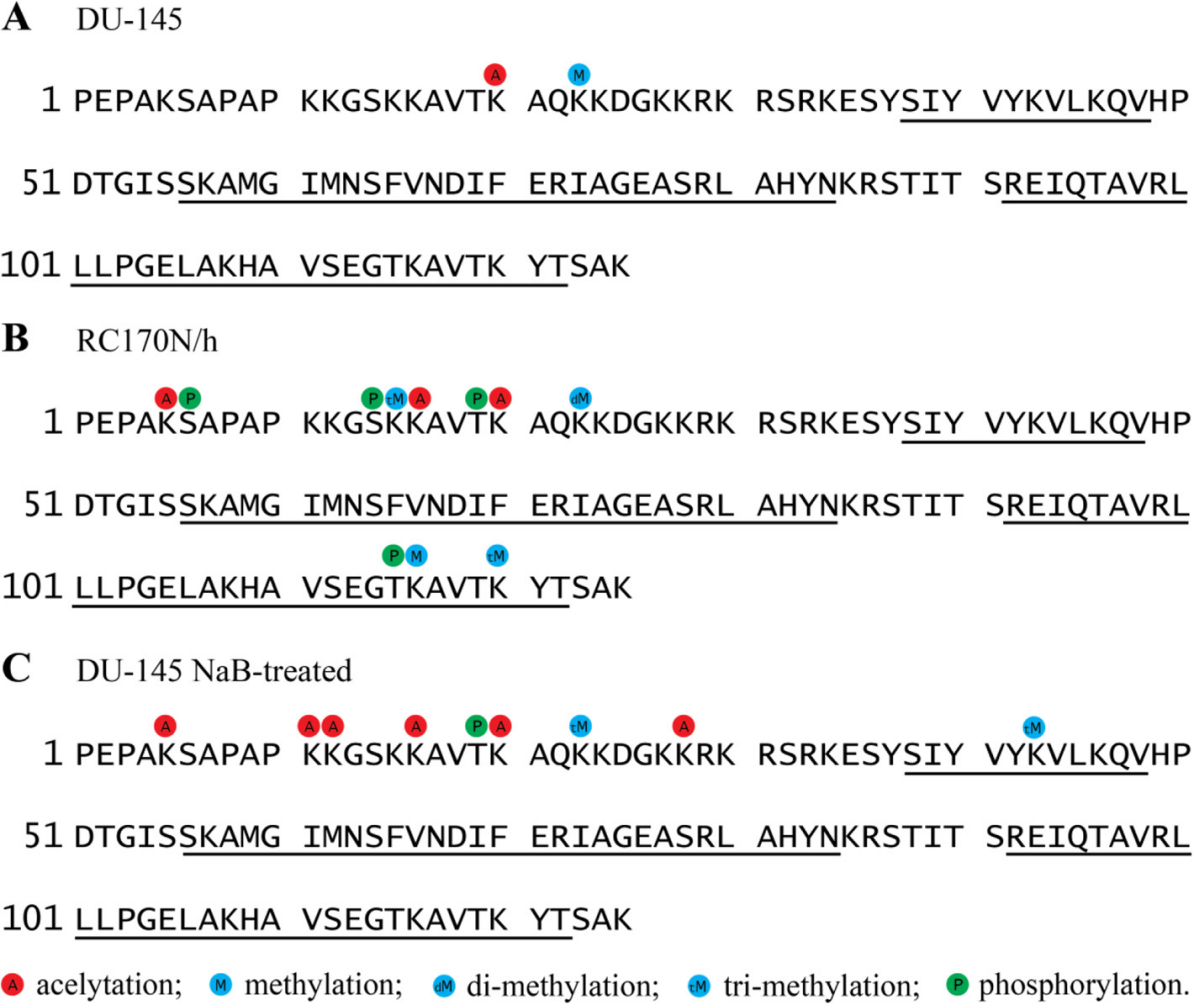
Post-translational modifications on the histone H2B. **a** The acetylation and methylation sites of H2B in the human prostate cancer DU-145 cells. **b** The acetylation, methylation, and phosphorylation of H2B in the non-malignant prostatic RC170N/h cells. **c** Histone H2B modifications in the DU-145 cells after sodium butyrate treatment.  indicates acetylation,  indicates methylation,  indicates di-methylation,  indicates tri-methylation, and  indicates phosphorylation. The H2B histone sequence is presented at the lower part of the figure. The underscored sequences represent the alpha helices in the structured domains of the histone

The histone proteins H3 and H4 hetero-tetramer are flanked on each side by an H2A and H2B hetero-dimer, with H3-H4 and H2A-H2B each interacting with different parts of the nucleosomal DNA. Aberrant activities of DNA methyltransferases and acetyltransferases lead to epigenetic remodeling of chromatin and have been implicated in carcinogenesis [1–3]. At this time, not much is known about the post-translational modifications of histones other than H3 in prostate cancer cells. Recent studies in yeast have revealed the importance of H2B in transcriptional regulation [4]. In this study, post-translational modifications of histone H2B from the human prostate cancer cell line DU-145 and the non-malignant prostatic cell line RC170N/h were analyzed by liquid chromatography-mass spectrometry (LC-MS/MS) (see Additional file 1) [5–7]. The status of H2B acetylation in DU-145 cells was illustrated in Fig. 1a, with acetylation determined on a single lysine (K) residue at amino acid sequence position 20 (K20). Lysine at position 23 (K23) was found to be di-methylated as shown in Fig. 1a. The acetylation at K20 and di-methylation at K23 were observed on the tryptic peptides, ^16^KAVTKAQK^23^ and ^17^AVTKAQKKDGKK^28^.

Analyses of H2B from RC170N/h cells revealed acetylation at K5, K16, and K20 (Fig. 1b). The methylations were found to be tri-methylated at K15 and K120, di-methylated at K23, and mono-methylated at K116 (Fig. 1b). The same peptides ^16^KAVTKAQK^23^ and ^17^AVTKAQKKDGKK^28^ that were analyzed for the DU-145 cells, together with other peptides including ^1^PDPSKSAPAPKKGSKKAVTKAQK^23^ and ^109^HAVSEGTKAVTK^120^, were examined for these modifications. The non-malignant RC170N/h cells clearly had more acetylated and methylated lysine residues on H2B than the DU-145 cancer cells.

To evaluate the histone deacetylase (HDAC) activity, DU-145 cells were treated with sodium butyrate, an inhibitor of HDACs. After butyrate treatment of these cells, acetylation on lysine residues, K5, K11, K12, K16, K20, and K27 of H2B, was identified (Fig. 1c). Specifically, the acetylation changes were detected in the following peptides, ^6^SAPAPKKGSK^15^, ^16^KAVTKAQK^23^, ^1^PEPAKSAPAPK^11^, and ^17^AVTKAQKKDGKK^28^. The fact that acetylation on H2B in the DU-145 cells was detected in multiple additional lysine residues after HDAC inhibition by sodium butyrate suggests that there was an excessive HDAC activity in the DU-145 cells. Acetylation of K5, K16, and K20 was also observed in the non-malignant RC170N/h cells (Fig. 1b, c). These data showed that the DU-145 cancer cells had a single K20 acetylation site, compared to the non-malignant RC170N/h cells that had three sites at K5, K16, and K20. The NaB-treated DU-145 cells had six acetylation sites at K5, K11, K12, K16, K20, and K27. The differences in the acetylation sites were detected at the N termini, without involving the alpha helices which start at amino acid residue 37 of H2B (Fig. 1, underscored sequences). The small lung carcinoma cells have six sites at K5, K11, K12, K15, K16, and K20 [8]; The Jurkat cells have three sites at K12, K15, and K20 [9], whereas the untreated DU-145 cells in this study have one acetylated K20. These results indicate that there are clear differences in acetylation sites among the human cell lines. These differences constitute the epigenetic signatures of individual neoplastic clones.

**Fig. 2:**
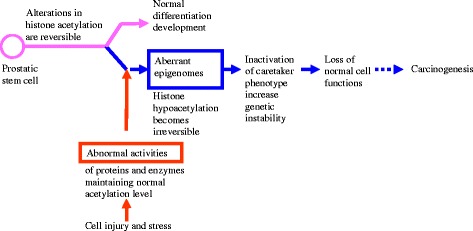
Hypothetical pathways of carcinogenesis from prostatic stem cells. Histone hypoacetylation leads to disruption of the normal epigenome in prostatic stem cells. The aberrant epigenome with hypoacetylation may be established when the reversible alterations in acetylation become irreversible. This is due to the abnormal histone deacetylase activities. As a result, caretaker phenotype and critical genes are inactivated. These eventually lead to carcinogenesis

We next examined changes of the H2B methylation status in the DU-145 cells upon HDAC inhibition. After sodium butyrate treatment, additional methylation on K43 was found (Fig. 2c), as compared to only K23 methylation in the untreated DU-145 cells (Fig. 1a).

There was no obvious phosphorylation detected on histone H2B peptides from the DU-145 prostate cancer cells. However, phosphorylation was detected at serines S6 and S14, as well as threonines T19 and T115 of the H2B histone in the non-malignant prostatic RC170N/h cells (Fig. 1b). Phosphorylation was detected on H2B peptides, ^16^KAVTKAQK^23^, ^1^PDPSKSAPAPKKGSKKAVTKAQK^23^, and ^109^HAVSEGTKAVTK^120^. When the DU-145 cells were treated with sodium butyrate (NaB), phosphorylation was found on threonine T19 (Fig. 1c). The increase in phosphorylation of H2B in DU-145 cells after NaB treatment is particularly intriguing. This provides the first evidence of the possible cross talk of HDACs and serine/threonine kinases.

As illustrated in Fig. 2, we postulate that the abnormal HDAC activity could turn the reversible histone modifications into irreversible, leading to perpetual aberrant epigenomes. An enhanced HDAC activity or a reduced K-acetyltransferase activity would tip the balance towards deacetylation. These could perpetuate the epigenetic changes and result in carcinogenesis. Targeting epigenetic modifications by inhibiting HDACs and DNA methyltransferases has become novel cancer therapies [2, 3, 10–12]. This study also suggests that HDAC inhibitors may be a potential therapeutic option for prostate cancer.

In conclusion, the histone H2B of DU-145 prostate cancer cells are hypoacetylated, hypomethylated, and dephosphorylated. Histone deacetylase inhibitor reversed this phenotype. Epigenetic agent may therefore be useful for prostate cancer therapy and worth further investigation.

## Additional file

Additional file 1:
**Materials and Methods** **[** 3 **,** 6 **,** 13 **–** 16 **].** (DOCX 26 kb)

## Abbreviations

HDAC: histone deacetylase
K: lysine
NaB: sodium butyrate
S: serine
T: threonine
