# Supplementary material for: Hypoacetylation, hypomethylation, and dephosphorylation of H2B histones and excessive histone deacetylase activity in DU-145 prostate cancer cells
Source: J Hematol Oncol. 2016 Jan 12;9:3. doi: 10.1186/s13045-016-0233-x (PMC4709959; doi:10.1186/s13045-016-0233-x)
Supplement: Additional file 1: — Materials and Methods [ 3 , 6 , 13 – 16 ]. (DOCX 26 kb) [file 13045_2016_233_MOESM1_ESM.docx]

**Methods**

***Cell cultures***

Human prostate cancer cell line DU-145 was maintained in RPMI-1640 supplemented with 10% heat-inactivated fetal calf serum. Cultures were exposed to 2 mM sodium butyrate for one day in studies to determine the epigenetic effect of butyrate on the cell lines. A telomerase-immortalized non-malignant human prostate epithelial cell line RC170N/h clone 7 (RC170N/h) was maintained in a serum-free keratinocyte medium supplemented with bovine pituitary extract and recombinant epidermal growth factor (Life Technologies, Inc) [3, 6, 13, 14]. The RC170N/h cell line was kindly provided by Dr. John S. Rhim, Uniformed Services University of the Health Sciences, Bethesda, MD.

***Histone isolation***

Lysates of cultured cells were prepared in the presence of sodium butyrate, a HDAC inhibitor, as previously described [15]. Histones were isolated by using a histone purification kit (Active Motif). Briefly, the histone binding resin was added to the lysates, and sequential elution of the resin was done to separate the H2A and H2B containing fractions from H3 and H4. The H2A-H2B fractions were further separated on SDS-PAGE gel, and the gels were subsequently stained with 0.5% Coomassie blue G-250. These gels were then treated with methanol-acetic acid and finally Milli-Q water for destaining. The H2B bands that migrated slower than H2A were excised for further analyses. The H2B bands were minced into 1 x 1 mm pieces, destained with 50% acetonitrile/25 mM ammonium bicarbonate, dehydrated with 100% acetonitrile, and finally dried in a vacuum centrifuge. Approximately 5-10 pmol quantity of the H2B histones was used for further analysis of post-translational modifications by mass spectrometry as described below.

***In-gel digest and LC-MS/MS analysis***

In-gel trypsin digestion of the excised H2B protein band was performed as previously described [16]. Briefly, the gel pieces were treated with 20 mM dithiothreitol for 45 min at 55^o^ C, and then incubated with 55 mM iodoacetamide at RT in dark for 30 min. The reduced and alkylated proteins were digested with a trypsin solution at 10 ng/µl in 25 mM ammonium bicarbonate (pH 8.0) overnight at 37^o^C. The peptides were extracted twice with 0.1% trifluoroacetic acid and 0.1% trifluoroacetic acid/50% acetonitrile, respectively before being dried in a vacuum centrifuge. The volumes were adjusted to 15 µl with 0.1% trifluoroacetic acid, of which 5 µl was loaded for LC-MS/MS analyses (see below).

The LC-MS/MS analysis was performed in the proteomic core service lab at the Rockefeller University, NY, as previously described . Briefly, 5 µl of trypsin-digested H2B peptides was separated by a 66-min gradient elution with the Dionex capillary nano-HPLC system (Dionex, Sunnyvale, CA) at a flow rate of 0.25 µl/min that is directly interfaced with a Thermo-Fisher LTQ-Obritrap mass spectrometer (Thermo Fisher, San Jose, CA). The analytical column was a fused silica capillary column with C-18 resin. The mobile phase A was consisted of 0.1% formic acid in water, and mobile phase B was 0.1% formic acid in acetonitrile. The LTQ-Orbitrap mass spectrometer was operated in the data-dependent acquisition mode using the Xcalibur 2.0.7 software (Thermo Fisher, San Jose, CA). The experiment consisted of a single full-scan mass spectrum in the Orbitrap (400-1,800 m/z, 30,000 resolutions), followed by six data-dependent MS/MS scans in the ion trap at 35% normalized collision energy. The dynamic exclusion parameters were as follows: repeat count=1, repeat duration = 30 ms, exclusion list = 100 ms and exclusion time= 90 ms. The acquired spectra (Thermo .raw files) were converted to .mgf files and searched against Uniprot Human database (*Homo sapiens*) for peptide identification using Mascot (Matrix Science Ltd., Boston, MA). The following Mascot search parameters were used: enzyme, trypsin; static modification, carbamidomethyl (C); dynamic modifications, acetylation (K), methylation (K), dimethylation (K), trimethylation (K), and phosphorylation (ST); peptide mass tolerance, 10 ppm; fragment ion mass tolerance, 0.5 Da. To increase the confidence of peptide identification, only peptides with a Mascot ion score greater than 15 were used for further analysis.

3. Cang S, Feng J, Konno S, Han L, Liu K, Sharma SC, Choudhury M, Chiao JW: **Deficient histone acetylation and excessive deacetylase activity as epigenomic marks of prostate cancer cells.** *Int J Oncol* 2009, **35:**1417-1422.

6. Gu Y, Li H, Miki J, Kim KH, Furusato B, Sesterhenn IA, Chu WS, McLeod DG, Srivastava S, Ewing CM, et al: **Phenotypic characterization of telomerase-immortalized primary non-malignant and malignant tumor-derived human prostate epithelial cell lines.** *Exp Cell Res* 2006, **312:**831-843
